# War Exhibit at the Recent Meeting of the A. M. A.

**Published:** 1919-09

**Authors:** 


### WAR EXHIBIT AT THE RECENT MEETING OF THE A. M. A.

At the recent meeting of the American Medical Association at Atlantic City, June 9 to 14, an exhibit, prepared under the authority of the Secretary of War and the Surgeon-General, by the curator of the Army Medical Museum, Washington, D. C., was on view, which illustrated the advances attained by the medical department of the army during the late war in its control of sanitation, medicine, surgery, hospitalism, and reconstruction of the disabled.

Construction and administration of hospitals for the care of sick and wounded were demonstrated by the Hospital Division, with typical layouts of hospitals and views of scenes at a number of hospitals.

The Sanitation Division operated a "delousing" plant, to show how typhus fever was kept out of the country by thoroughly cleaning the men and their clothing of the "cootie." Practical sanitation of army posts and mobile units, both at home and abroad, were shown by a complete series of models of field sanitary appliances designed and constructed at the Laboratory of Field Sanitation, Ft. Riley, Kansas.

Methods employed by the army in handling infectious diseases, which have resulted in a marked reduction of the chief sick rates in the preceding generation, were exhibited by the section of epidemiology of the Laboratory Division. It included a complete field-laboratory outfit, such as was used on the western front for making diagnoses of suspected epidemics of disease, so that prompt isolation and treatment could be applied.

Lecture-slides, placards, posters, and pamphlets used by the army in combating venereal diseases during the training of the army and also during the demobilization, to interest soldiers returning to civilian life in the fight against this disease, also were shown.

Wax and plaster models were on view under the Division of Surgery, showing the results of operations upon wounds of the face and limbs by various methods, and demonstrating the fine results that have followed these measures in the case of which men, who, having practically lost their entire faces, have been given new ones. There were also models illustrating the effects of bone grafting in shell-wound cases.

Under the Orthopedic-Surgery Section was shown the attention given to the feet of the soldier. The exhibit embraced foot inspection, shoe alterations, foot exercises, and simple exercising-apparatus. The application of splints and the fitting of wooden legs, provisional arms, and special appliances, such as hooks, and tool-holders for those that have lost arms also was shown.

An X-ray machine which generates its own power for operation, and a bedside unit, together with an X-ray ambulance, packed with everything necessary for radiographic work in the field, with the operating crew, demonstrated this feature of army service.

The care and treatment of insane and neurotic patients were shown in an exhibit of the section of neuropsychiatry. The work of the army in caring for its horses and mules was also depicted in an exhibit of the Veterinary Division.

The Division of Physical Reconstruction had an interesting exhibit, illustrating the work of furnishing therapeutic adjuncts to medical and surgical treatment, with a view to hastening recovery and securing the best functional restoration for the disabled soldier. Articles of workmanship turned out by these patients, including beautiful knitted bags, knotted cord-work, bead necklaces, woven belts, colonial mats, and poster work at the various hospitals were shown.

Apparatus from the Medical-Research Laboratory at Hazelhurst Field, Mineola, New York, had been sent by the Air Medical Service, to illustrate the manner of training fliers so as to habituate themselves to motion in the air. It included types of oxygen-supply apparatus for use while flying at high altitudes; the whirling chair for equilibrium tests; apparatus for testing judgment of distances, testing the eyes, testing shade differences, and other tests. A rebreathing-apparatus for the altitude classification of aviators also was shown.

The work of publication of newspapers and magazines at army hospitals by and for soldier patients was illustrated by an exhibit.

The first American flag to lead a United States military detachment into Europe during the war also was exhibited. The flag was carried by the first army contingent to leave the United States, May 8, 1917.—*Clinical Medicine.*

